# Exploring Gender-Specific Correlations Between Nutritional Intake, Body Composition, Psychological Skills, and Performance Metrics in Young Taekwondo Athletes

**DOI:** 10.3390/nu17071202

**Published:** 2025-03-29

**Authors:** Mohammad Hossein Samanipour, Mohammad Azizi, Omid Salehian, Halil Ibrahim Ceylan, Juan Francisco Mielgo-Ayuso, Juan Del Coso, Raul Ioan Muntean, Nicola Luigi Bragazzi, Tomás Herrera-Valenzuela

**Affiliations:** 1 Department of Sport Science, Imam Khomeini International University, Qazvin 34148-96818, Iran; samani.mh@gmail.com; 2 Faculty of Physical Education, Payam Noor University, Karaj 319, Iran; mohammad.azizi.1355.30@gmail.com; 3 Department of Sport Nutrition and Fitness, Applied and Science University, Tehran 13114-16846, Iran; salehian.omid@gmail.com; 4 Department of Physical Education of Sports Teaching, Faculty of Sports Sciences, Atatürk University, 25240 Erzurum, Türkiye; 5 Department of Health Sciences, Faculty of Health Sciences, University of Burgos, 09001 Burgos, Spain; jfmielgo@ubu.es; 6 Sport Sciences Research Centre, Rey Juan Carlos University, 28943 Fuenlabrada, Spain; juan.delcoso@urjc.es; 7 Department of Physical Education and Sport, Faculty of Law and Social Sciences, University “1 Decembrie 1918” of Alba Iulia, 510009 Alba Iulia, Romania; 8 Laboratory for Industrial and Applied Mathematics (LIAM), Department of Mathematics and Statistics, York University, Toronto, ON M3J 1P3, Canada; robertobragazzi@gmail.com; 9 Department of Physical Activity, Sports and Health Sciences, Faculty of Medical Sciences, Universidad de Santiago de Chile (USACH), Santiago 8370003, Chile; tomas.herrera@usach.cl

**Keywords:** combat sports, athletic performance, sports nutrition, psychological resilience, high-intensity training, youth athletes

## Abstract

**Objectives:** Taekwondo performance is influenced by a complex and dynamic interplay of physical, nutritional, and psychological factors, all of which contribute to competitive success. However, the gender-specific relationships among these factors in young high-performance athletes remain understudied. This study aimed to fill in this knowledge gap. **Methods**: A cross-sectional study was conducted with 35 elite taekwondo athletes (male: *n* = 20, female: *n* = 15, age: 13 ± 1 years). Participants underwent anthropometric assessments, dietary evaluations, and psychological skill assessments during an 8-week training camp before the World Taekwondo Championships. Physical performance was assessed using the Frequency Speed of Kick Test (FSKT_mult_) and the Taekwondo-Specific Agility Test (TSAT). Statistical analyses included independent t-tests, correlation analyses, and regression models. **Results**: Males exhibited significantly higher fat-free mass (FFM: 42.8 ± 2.9 kg vs. 36.3 ± 1.6 kg, *p* < 0.001), skeletal muscle mass (SMM: 31.1 ± 2.2 kg vs. 28.2 ± 1.6 kg, *p* < 0.001), and energy intake (32.4 ± 4.6 kcal/kg vs. 29.3 ± 3.1 kcal/kg, *p* = 0.032) than females. Males also had greater dietary intakes of vitamin A, vitamin C, magnesium, and iron (all *p* < 0.05). There were no gender differences in any psychological attributes associated with emotional intelligence, sport success perception, and mental toughness. Although the total kick count in the FSKT_mult_ was similar for male and female taekwondo athletes (100.2 ± 4.6 vs. 97.5 ± 5.9 kicks, *p* = 0.139), males outperformed females in round 4 (19.4 ± 1.1 vs. 18.6 ± 1.4 kicks, *p* = 0.048) and round 5 (18.2 ± 1.0 vs. 17.2 ± 1.0 kicks, *p* = 0.007) of this test, suggesting higher physical performance maintenance during the test. Regression models indicated that body mass (β = 0.901, *p* < 0.001) and calcium intake (β = 0.284, *p* = 0.011) predicted performance in males, while body mass (β = 1.372, *p* < 0.001), protein intake (β = 0.171, *p* = 0.012), and emotional regulation (β = 0.174, *p* = 0.012) were key predictors in females. **Conclusions**: These findings highlight the importance of an integrated approach to training, nutrition, and psychological preparation in optimizing taekwondo performance. While males and females demonstrated similar psychological resilience and total kick output in a taekwondo-specific test, males exhibited superior endurance in later test rounds of this test. Performance optimization in young elite taekwondo athletes may require the implementation of gender-specific training and nutrition strategies, emphasizing body weight control and calcium intake for males and protein intake for females.

## 1. Introduction

Taekwondo (TKD) is a dynamic and demanding combat sport that requires a balance of physical, technical, and psychological readiness. The nutritional status of TKD athletes is crucial for enhancing performance, especially given the demands of training and competition, which often take place under stressful conditions [1,2]. Therefore, to achieve competitive success, TKD practitioners must prioritize their nutritional requirements, ensuring adequate energy intake and proper hydration [3]. Equally important is optimal mental preparation, which involves setting clear goals, adhering to personal plans, minimizing distractions, maintaining positivity, and managing emotions [4,5]. In TKD, the development of technical skills and physical fitness is paramount, alongside the cultivation of psychological well-being, discipline, self-control, and mutual respect [6,7]. These factors are important for TKD athletes of all age groups, including young athletes.

Beyond physical prowess, TKD instills a “fighting spirit” characterized by inner strength, emphasizing courtesy, personal growth, strict self-discipline, and conflict resolution rather than aggression to navigate challenges effectively and foster a positive mindset [8]. This holistic approach highlights the interconnected nature of athletic success in TKD, where the various factors work synergistically to enhance performance, optimize recovery, prevent injuries, and mitigate the physical and mental stress of high-intensity competition [9,10,11].

Overall, nutrition plays a vital role in maintaining a healthy lifestyle for individuals, including adolescent athletes and non-athletes [12,13,14]. The American College of Sports Medicine, the American Dietetic Association, and Dietitians of Canada have reported that optimal nutrition enhances physical activity, performance, and recovery after exercise [15]. For TKD athletes, adequate nutrient intake, including carbohydrates (CHO), proteins, fats, vitamins, and minerals, is essential for supporting physiological processes, muscle repair, and cognitive and immune functioning, thereby optimizing athletic performance and resilience to stress [16]. Enhancing nutritional status and fostering positive attitudes towards healthy dietary practices are essential for the overall well-being of TKD athletes [11].

For instance, a study on male TKD athletes investigated the nutritional intake and body composition during two weeks of weight management [17], finding that their habitual and pre-competition diets were suboptimal, with energy, total carbohydrate, calcium, and water intakes below recommended levels, while fat and salt consumption exceeded recommendations. Therefore, in addition to training programs that cultivate both the psychological resilience and physical capabilities of TKD athletes, well-structured nutritional strategies are crucial.

In addition to nutrition and psychological preparation, TKD performance relies on specific physical assessments to measure sport-specific endurance and power [18]. The ATP-PCr system represents the main source of energy during high-intensity attack actions in TKD matches [19]. Additionally, the glycolytic system supports the maintenance of these actions when repeated techniques are being performed during a match [20]. Incorporating specific physical assessments throughout the season is fundamental for evaluating and enhancing TKD performance in high-performance athletes. The Frequency Speed of Kick Test (FSKT_mult_) serves as a vital assessment tool for assessing kicking endurance and fatigue resistance among athletes. This test quantifies performance by measuring the total number of kicks executed in 10 s kicking bouts and by calculating the kick decay index (KDI), which indicates kicking performance decline over successive bouts. Research has demonstrated its effectiveness in providing insights into athletes’ endurance capabilities and their ability to maintain performance under fatigue [21,22].

A study by Santos and Franchini [21] highlighted that TKD athletes undergoing regular FSKT_mult_ assessments showed significant improvements in their kicking endurance over time, correlating with enhanced overall performance in competitions. Another investigation by Ulupinar et al. [22] found that athletes who consistently trained using the insights gained from FSKT_mult_ not only improved their kicking speed but also exhibited greater resilience during matches, as indicated by their ability to maintain technique and power despite fatigue. In addition, Santos and Franchini [23] explored the FSKT performance among female TKD athletes at different competitive levels. The results indicated that the International/National group significantly outperformed the State/Regional group in both the FSKT 10s and FSKT_mult_ assessments, highlighting the effectiveness of this test in discriminating against the competitive level of trained TKD athletes. Accordingly, specific physical assessments like the FSKT_mult_ provide critical data that can inform tailored training strategies aimed at enhancing both physical and psychological readiness for competition. In this sense, TKD athletes perform short and dynamic actions at different joint ranges, generally accelerating limbs until they reach the final range of motion or the opponent [24].

Considering the studies discussed above, the relationship between physical, psychological, and nutritional factors plays a pivotal role in TKD performance [25,26]. However, despite the clear influence of these interconnected elements, limited research has examined their combined impact [27]. Additionally, few studies have comprehensively examined these variables in a gender-specific context within TKD. Understanding how these factors interact differently for male and female TKD athletes could provide valuable insights into optimizing training and performance outcomes in this martial art [28].

This study aimed to fill in this knowledge gap by examining the differences in body composition, psychological skills, nutrient intake, physical performance, and their correlations in a sample of young male and female TKD athletes. We hypothesized that male and female TKD athletes would exhibit distinct differences in the variables under study, with unique correlations, influencing their overall athletic success.

## 2. Materials and Methods

### 2.1. Study Design

This cross-sectional study was conducted on top-performing young male and female TKD athletes preparing for the 2023 World Taekwondo Championships in Sarajevo, Bosnia and Herzegovina. The study period lasted for 8 weeks and coincided with the competitive training phase, designed to optimize peak performance for the championship, including technical–tactical and physical conditioning training (around 20 h/week). Each day consisted of 2–3 training sessions, and all athletes followed an identical training regimen, which included estimated food intake assessments and anthropometric measurements throughout the training camp. Nutritional habits were assessed over three days, encompassing both weekdays and weekends, to ensure accuracy. The final assessments were conducted during the last block of the training camp, just before the competition, focusing on nutritional intake (macro/micronutrients), psychological skills, and sport-specific performance.

The training followed a structured eight-week preparatory model. All evaluations and training sessions took place at the Iran Taekwondo Federation’s Taekwondo House in Tehran. All evaluation sessions were conducted under controlled environmental conditions, with the temperature maintained at 22–25 °C and humidity at 41–55%, ensuring consistency across assessments. Testing was scheduled at the same time of day (10 a.m. ± 2 h) to minimize the influence of circadian rhythms and reduce the likelihood of reporting errors. Participants were instructed to refrain from consuming any ergogenic aids (e.g., pre-workout supplements, caffeine, creatine, beta-alanine, protein supplements, etc.) for at least four weeks before the study to eliminate potential confounding effects. Additionally, they were required to maintain proper sleep hygiene throughout the training camp and avoid altering their dietary habits during the experimental period. FSKT_mult_ measurements were performed between 9:00 a.m. and 1:00 p.m., and after recovery, psychological tests were made with silence and without tension between 4:00 p.m. and 5:00 p.m.

### 2.2. Participants

A total of 40 Iranian National team TKD athletes were recruited for this study, including 20 males (age: 13 ± 1 years; body mass: 51.5 ± 17.1 kg; height: 165 ± 20 cm; international competition experience: 1 year) and 15 females (age: 13 ± 1 years; body mass: 46.1 ± 15.0 kg; height: 153 ± 15.0 cm; international competition experience: 1 year). Initially, five additional female athletes were considered for participation; however, they were excluded due to technical reasons before enrollment in the study. All athletes in this study were free of neuromuscular injuries or disorders at the time of the study. All participants were actively engaged in a structured training program consisting of two macrocycles per year, specifically designed to optimize their preparation for the world championships and major tournaments. Participants had previously competed in the 2022 World Taekwondo Championships, collectively earning 2 gold, 2 silver, and 1 bronze medals. Following their participation in the 2023 World Taekwondo Championships, the male athletes secured 1 gold and 2 bronze medals, while the female athletes achieved 3 gold and 3 silver medals. Participants were categorized as Tier 4–5 according to the participant classification framework by McKay et al. [29].

Before participation, all athletes and their legal guardians were fully informed about the study procedures and provided written assent and informed consent, respectively, authorizing the use of their data for scientific purposes. This study was approved by the Institutional Ethics Committee of the Sport Science Research Institute of Iran (IR.SSRC.REC.2409-2822).

### 2.3. Body Composition and Anthropometric Measurement

During the training camp, body weight was measured using a calibrated digital scale (SECA 803, Hamburg, Germany) with a precision of ± 0.1 kg, while height was assessed with a stadiometer (SECA 206, Hamburg, Germany) following standardized protocols as described by [30]. Body mass index (BMI, kg/m^2^) was calculated by dividing body weight (kg) by the square of height (m^2^). To ensure accuracy and consistency, all measurements were conducted in the morning, after an overnight fasting period, with participants dressed in lightweight clothing and barefoot. Body composition (BC) assessment was performed using the InBody 720 (Biospace Co., Ltd., Seoul, Republic of Korea), as previously suggested [31]. The InBody 720 is a device known for its high test–retest reliability, with an Intraclass Correlation Coefficient (ICC) of 0.9995, ensuring precise and reproducible measurements [32,33]. The assessed BC variables included fat mass (FM), fat-free mass (FFM), body fat percentage (%BF), and skeletal muscle mass (SMM).

### 2.4. Psychological Skills Questionnaires

#### 2.4.1. Emotional Intelligence Scale

The “Emotional Intelligence Scale” (EIS) was initially developed by Schutte et al. [34], following the model by Salovey and Mayer [35], and subsequently validated by Lane et al. [36,37]. In the sports arena, EIS is a self-assessment questionnaire consisting of 33 items, three of which (5, 28, and 33) are reverse-scored, and six factors that show the individual’s desire and persistence to continue participating in sports activities. The six factors in this questionnaire include: (I) evaluating other people’s feelings (seven items), (II) evaluating one’s feelings (five items), (III) self-regulation (five items), (IV) social skills (five items), (V) using emotions (seven items), and (VI) optimism (four items). Items are presented in the form of declarative sentences and questions, and each is answered on a five-point Likert scale, ranging from strongly disagree—1 to strongly agree—5, and scored as 2 = disagree, 3 = neutral, and 4 = agree [34].

The average score can range from 33 to 165, with higher scores indicating more characteristics of emotional intelligence. The mean emotional intelligence score is approximately 124 (with a standard deviation of about 13), with scores < 111 or >137 being considered unusually low or high [38]. Lane et al. [36] provided a comprehensive overview of the questionnaire’s development, using two studies to present its content validity and confirmatory factor analysis (CFA) of the subscales. In addition, Eydi et al. [39] provided evidence of its factor analysis and reliability in a translated version for those who speak Farsi. In the present study, CFA could not be calculated due to small sample sizes; however, Cronbach’s alpha coefficients ranged between 0.75 and 0.87 across subscales, and the coefficient was 0.80 overall. Although Lane et al. [40] suggested removing 14 items (like “I find it hard to understand the non-verbal messages of other people” and “When I am faced with obstacles, I remember times I faced similar obstacles and overcame them”) that lacked emotional content, the original 33-item version was employed in the present investigation, being the version validated by Eydi et al. [39].

#### 2.4.2. Sport Success Scale (SSS)

The “Sport Success Scale” (SSS) was designed based on a robust conceptual framework devised by drawing from the theory of dynamical systems, the multi-stage theory of motor learning, and the motivation theory, and standardized by Mousavi and Vaez Mousavi [41]. This tool consists of 29 questions that measure flow state, attention, technique, sensitivity to error, commitment, and achievement. This scale is set on a six-point Likert scale, ranging from strongly disagree—1 to strongly agree—6. Reliability (Cronbach’s alpha coefficients) was estimated as follows: flow state 0.89, attention 0.88, technique 0.89, sensitivity to error 0.88, commitment 0.89, achievement 0.89, and overall alpha coefficient 0.89. In addition, the content-related validity and structure-related validity were confirmed to be satisfactory [41].

#### 2.4.3. Sport Mental Toughness Questionnaire (SMTQ)

This questionnaire was designed to measure mental toughness with three subscales: confidence, constancy, and control [42,43]. The tool includes 14 questions that measure three subscales: confidence (six questions), constancy (four questions), and control (four questions). Each question has four answer options, from completely incorrect and almost incorrect to almost and completely correct. Details on score calculations are provided elsewhere [42,43]. The validity and reliability of this questionnaire were confirmed by Sheard and collaborators [43]. The Cronbach’s alpha coefficient of confidence, constancy, and control was reported to be 0.80, 0.74, and 0.71, respectively [43]. The Sport Mental Toughness Questionnaire (SMTQ) is the only valid and reliable tool in the field of mental toughness that includes emotion control and negative energy, which have been identified as critical characteristics of successful athletes [44,45,46,47]. Furthermore, this scale was evaluated and applied in research by Nabilpour et al. [48], specifically on top elite male TKD athletes, further supporting its relevance and applicability in combat sports. As athletes were adolescents, two experts in sport psychology and sport physiology provided detailed explanations to clarify the questions not fully understood by the participants.

### 2.5. Taekwondo-Specific Performance

Previous studies have demonstrated high test–retest reliability for the FSKT_mult_. For example, Santos and Franchini [21] reported ICCs ranging from 0.63 to 0.83, indicating good reliability across different performance measures. In addition to its reliability, the FSKT_mult_ has exhibited strong construct validity. Santos and Franchini [23] further demonstrated that the test effectively differentiates between female TKD athletes of varying competitive levels, reinforcing its utility as a performance assessment tool in the sport. The FSKT_mult_ was conducted following standardized protocols, as previously described in the literature, ensuring consistency and comparability across studies [49,50].

Performance was determined by the total number of kicks (total kicks) and the KDI. The Taekwondo-Specific Agility Test (TSAT) was measured according to previously established protocols, ensuring standardized assessment procedures for agility performance in TKD athletes. This test is designed to evaluate movement speed, reaction time, and change of direction ability, which are critical components of combat effectiveness in TKD. Following recommended methodologies, athletes performed the TSAT under controlled conditions, with standardized starting positions, timing mechanisms, and movement sequences to ensure reliable and reproducible results [51]. The time required to complete the TSAT was used as a performance outcome and was assessed with an electronic timing system (Brower Timing Systems, Salt Lake City, UT, USA). Each athlete was given three trials, with the best performance recorded for analysis. All the performance tests were conducted indoors on a taekwondo tatami, ensuring a consistent and sport-specific testing environment.

Before testing, athletes completed a structured 10 min warm-up, consisting of 5 min of running, followed by dedicated time for static and ballistic stretching, as well as sport-specific submaximal exercises, including kicking, squatting, and jumping, to properly activate the neuromuscular system. To ensure familiarity and minimize learning effects, a three-day familiarization period was organized before the official testing sessions. Each test (FSKT_mult_ and TSAT) was performed on two separate days, allowing for adequate recovery and reducing potential fatigue-related effects on performance outcomes.

### 2.6. Nutrient Intake Measurement

Common methods used in research to assess food consumption include 24 h dietary recalls and Food Frequency Questionnaires (FFQ). However, the most widely used approach, despite its susceptibility to bias, is the food intake diary. Each of these methods has distinct advantages, and the choice of the assessment technique depends on the specific objectives of the study. Among these, the 24 h recall method is considered one of the most accurate techniques, as it provides a detailed estimation of food intake while minimizing bias, particularly underreporting. However, this method is subject to individual variations in memory recall and self-reporting accuracy, which can be influenced by mood, cognitive state, and physiological conditions throughout the day [52]. To reduce bias, particularly in younger athletes, participants in this study completed a structured 7-day open food report, documenting detailed information on the type and quantity of foods and fluids consumed [53].

This approach aimed to enhance the accuracy of dietary assessment, capturing habitual intake patterns more effectively while accounting for daily fluctuations in eating behavior [54]. Following data collection, nutritional information was entered into specialized software to analyze detailed macro- and micronutrient values, as outlined in the study by Samanipour et al. [31]. The analysis provided comprehensive insights into average energy intake (EI) and the distribution of macronutrients (proteins, carbohydrates, and fats), along with key micronutrients, including vitamins A, C, E, D, and B12, thiamine, riboflavin, niacin, folate, magnesium, zinc, calcium, and iron. This approach ensured a systematic and precise evaluation of dietary intake, facilitating a comprehensive assessment of athletes’ nutritional status [55].

### 2.7. Statistical Analysis

Descriptive statistics, including mean and standard deviation (SD), were used to summarize the data. The Shapiro–Wilk test was employed to assess the normality of the data distribution. Depending on the results of the normality tests, parametric tests (independent *t*-tests) or non-parametric tests (Mann–Whitney U tests) were used to compare variables between young male and female taekwondo athletes. For each comparison, effect sizes (ES) were computed using Cohen’s d, with values interpreted as small (0.2 ≤ d < 0.5), medium (0.5 ≤ d < 0.8), or large (d ≥ 0.8) effects, as per conventional benchmarks.

Pearson’s correlation coefficient was applied to evaluate the relationships between physical performance metrics and other variables (nutrient intake, anthropometric measurements, and psychological skills) for normally distributed data, while Spearman’s rank correlation coefficient was used for non-normally distributed data. Simple linear regression models were developed to explore the relationship between physical performance variables, specifically the total number of kicks in the FSKT_mult_ and the time from the TSAT, as dependent variables, while independent variables included anthropometric measurements, nutritional intake, and psychological skills. These stepwise regression analyses aimed to identify significant predictors for performance metrics. The Variance Inflation Factor (VIF) and tolerance were analysed to assess whether there was collinearity between the predictor variables. The aim was to detect possible multicollinearity problems in the regression models. The criteria used were that a VIF greater than 10 and a tolerance less than 0.1 would indicate high collinearity.

All statistical analyses were performed using GraphPad Prism (version 10.0, GraphPad Software, Inc., Boston, MA, USA) for Mac OS. Statistical significance for all tests was set at *p* < 0.05.

## 3. Results

Table 1 presents the anthropometric comparisons between young male and female TKD athletes, indicating no significant differences in height, body mass, or BMI between the two groups. However, significant differences were observed in BC parameters. Males exhibited a significantly higher FFM (42.8 ± 2.9 kg) compared to females (36.3 ± 1.6 kg, *p* < 0.001). Similarly, FM was significantly greater in males (3.5 ± 0.7 kg) than in females (2.9 ± 0.7 kg, *p* = 0.009). Additionally, males displayed a significantly higher body fat percentage (6.3 ± 0.4%) compared to females (5.6 ± 0.5%, *p* < 0.001). Lastly, SMM was significantly greater in males (31.1 ± 2.2 kg) than in females (28.2 ± 1.6 kg, *p* < 0.001), highlighting gender-related differences in muscle distribution and overall BC.

Table 2 presents the psychological attribute comparisons between young male and female TKD athletes, revealing no significant gender differences in emotional intelligence, social skills, or sports success (all *p* > 0.05). Similarly, no significant differences were observed in optimism or key mental toughness components, including confidence, control, and constancy, suggesting that both male and female athletes exhibit comparable psychological resilience and mental preparedness in the context of competitive TKD.

Table 3 reports nutrient intake among young male and female TKD athletes. Males had a significantly higher fat intake (1.4 ± 0.3 g/kg) compared to females (1.1 ± 0.1 g/kg; *p* = 0.003), as well as greater EI per kilogram of body weight (32.4 ± 4.6 kcal/kg vs. 29.3 ± 3.1 kcal/kg; *p* = 0.032). Regarding micronutrients, males consumed significantly more vitamin A (1386.8 ± 300.9 µg/day) than females (921.4 ± 323.8 µg/day; *p* < 0.001), vitamin C (102.0 ± 27.4 mg/day vs. 80.4 ± 20.0 mg/day; *p* = 0.014), magnesium (318.7 ± 45.1 g/day vs. 272.0 ± 41.7 g/day; *p* = 0.004), and iron (17.0 ± 2.4 mg/day vs. 14.4 ± 3.6 mg/day; *p* = 0.017). In contrast, females exhibited significantly higher vitamin B12 intake than males (6.7 ± 1.2 µg/day vs. 5.5 ± 1.5 µg/day; *p* = 0.017). 

Table 4 shows the results of the kick speed test and other performance metrics for male and female TKD athletes. No significant gender differences were observed in kick speed performance during rounds 1 to 3. However, males exhibited significantly better performance in round 4 (*p* = 0.048) and round 5 (*p* = 0.007) compared to females. Additionally, no significant gender differences were found in the total number of kicks, the KDI, or the TSAT, indicating comparable overall endurance and agility performance between male and female athletes, despite differences appearing in later rounds of the kick speed test.

**Figure 1:**
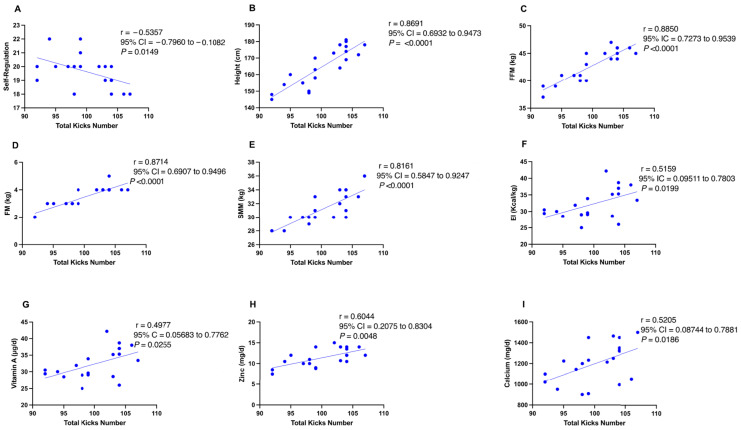
Pearson correlations between physical performance in the FSKT_mult_ and psychological skills and nutritional variables in young male taekwondo athletes. (**A**) Correlation between total kicks and self-regulation. (**B**) Correlation between total kicks and height. (**C**) Correlation between total kicks and fat-free mass (FFM). (**D**) Correlation between total kicks and fat mass (FM). (**E**) Correlation between total kicks and skeletal muscle mass (SMM). (**F**) Correlation between total kicks and energy intake (EI). (**G**) Correlation between total kicks and vitamin A intake. (H) Correlation between total kicks and zinc intake. (**I**) Correlation between total kicks and calcium intake.

Figure 1 presents the correlations between physical performance measured through the FSKT_mult_ and psychological attributes and nutritional variables in young male TKD athletes, highlighting key statistically significant relationships. The analysis indicated that body height, FFM, FM, SMM, EI, and the intakes of vitamin A, zinc, and calcium were positively correlated with the total number of kicks in the FSKT_mult,_ while self-regulation was negatively related with FSKT_mult_ overall performance (all *p* < 0.05).

**Figure 2:**
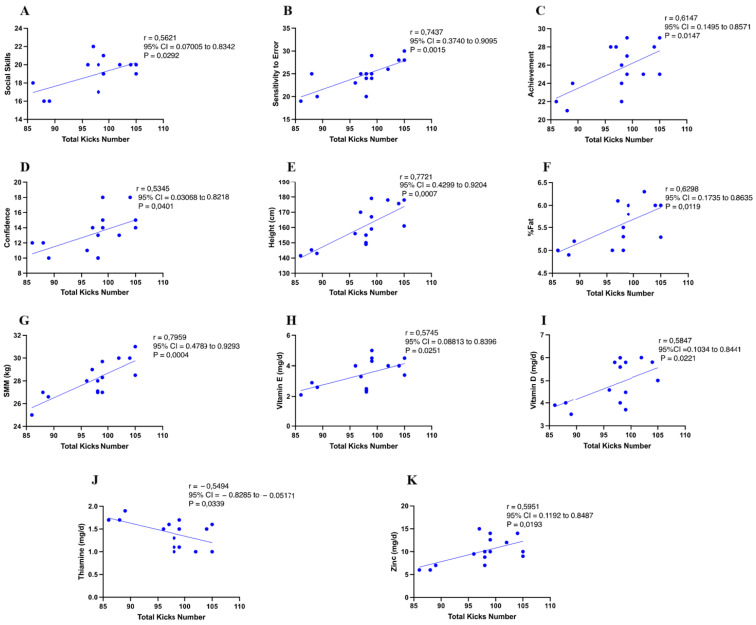
Pearson correlations between physical performance in the FSKT_mult_ and psychological skills and nutritional variables in young female taekwondo athletes. (**A**) Correlation between total kicks and social skills. (**B**) Correlation between total kicks and sensitivity to error. (**C**) Correlation between total kicks and achievement. (**D**) Correlation between total kicks and confidence. (**E**) Correlation between total kicks and height. (**F**) Correlation between total kicks and body fat percentage (%Fat). (**G**) Correlation between total kicks and skeletal muscle mass (SMM). (**H**) Correlation between total kicks and vitamin E intake. (**I**) Correlation between total kicks and vitamin D intake. (**J**) Correlation between total kicks and thiamine intake. (**K**) Correlation between total kicks and zinc intake.

Figure 2 presents the correlations between physical performance measured through the FSKT_mult_, psychological attributes, and nutritional variables in young female TKD athletes. The analysis indicated that social skills, sensitivity to error, achievement, and confidence were the psychological skills positively correlated with the total number of kicks in the FSKT_mult_ (all *p* < 0.05). The analysis also revealed that body height, %Fat, and SMM were the anthropometric variables positively correlated with the total number of kicks in the FSKT_mult_ (all *p* < 0.05).

Additionally, the intake of vitamins E, D, and zinc was positively correlated with the total number of kicks in the FSKT_mult_ (all *p* < 0.05), while the intake of thiamine was negatively correlated (*p* < 0.05). On the contrary, social skills, technique, confidence, and intakes of zinc and calcium were all negatively correlated with the TSAT (all *p* < 0.05), with BMI being the only variable positively correlated (*p* < 0.05). 

The best-fit regression model for predicting performance in young male TKD athletes included body mass (anthropometric variable), constancy (psychological variable), and calcium intake (nutritional variable) as key predictors (Table 5). The model showed that body mass was positively correlated with the number of kicks, with a non-standardized B coefficient of 0.417 (*p* < 0.001) and a standardized Beta coefficient of 0.901, indicating a strong association between weight and kicking performance (Table 5). On the other hand, constancy, a key psychological variable, exhibited a negative association with the total number of kicks, as indicated by a non-standardized B coefficient of −0.831 (*p* = 0.001) and a standardized Beta value of −0.358. This suggests that higher constancy (greater mental toughness) was linked to a lower number of kicks. Additionally, calcium intake emerged as a significant positive predictor, with a non-standardized B coefficient of 0.007 (*p* = 0.011) and a standardized Beta value of 0.284, indicating that higher calcium intake was associated with an increased number of kicks. This regression model accounted for 89.3% of the variance in the total number of kicks, with a standard error of 1.464, highlighting the strong predictive power of these variables in determining kicking performance.

It is important to note that the collinearity values were found to be within acceptable limits. Specifically, the tolerance values for body mass, constancy, and calcium intake were 0.660, 0.707, and 0.619, respectively, while the VIF values were 1.514, 1.414, and 1.616. These results indicate that there were no significant multicollinearity problems, as all predictors had tolerance values greater than 0.10 and VIF values less than 10, suggesting that they were not overly correlated with each other.

In Table 6, for females, the best-fit regression model for predicting performance included body mass and FFM (anthropometric variables), employing emotion (psychological variable), and protein, fat, and thiamine intakes (nutritional variables) as key predictors. Specifically, body mass was a significant predictor, with a non-standardized B coefficient of 0.966 (*p* < 0.001) and a standardized Beta of 1.372, suggesting a strong positive relationship between body weight and kicking performance. Dietary thiamine intake had a negative effect, with a non-standardized B coefficient of −6.296 (*p* < 0.001) and a standardized Beta of −0.327, indicating that higher thiamine intake was associated with fewer kicks in the FSKT_mult_. Protein intake (g/kg) had a positive effect, with a non-standardized B coefficient of 6.748 (*p* = 0.012) and a standardized Beta of 0.171. Additionally, FFM showed a negative relationship, with a non-standardized B coefficient of −2.551 (*p* = 0.001) and a standardized Beta of −0.683, suggesting that a higher FFM was associated with a lower number of kicks. Fat intake also had a negative association with kicking performance, with a non-standardized B coefficient of −19.049 (*p* = 0.001) and a standardized Beta of −0.269. Lastly, employing emotion, a psychological variable, was a direct predictor, with a non-standardized B coefficient of 0.676 (*p* = 0.012) and a standardized Beta of 0.174. This model explained 96.7% of the variance in the total number of kicks, with an error standard of 1.065.

Collinearity analysis revealed no significant problems in the regression model as none of the predictors exceeded the critical threshold of VIF = 10. Although body mass and FFM had the lowest tolerance values (0.137 and 0.147) and the highest VIF values (7.308 and 6.797), these remained within an acceptable range. The remaining variables, such as thiamine, protein, and fat intakes and emotion use, had high tolerance values and low VIFs, suggesting that multicollinearity did not affect the stability of the model or the interpretation of its predictors.

Regarding the TSAT, no significant variables were found to be predictive for males). However, in the model for females, dietary zinc intake (non-standardized B = −0.309, *p* = 0.001, standardized Beta = −1.164) had a significant inverse relationship with TSAT performance, suggesting that higher dietary zinc levels are associated with better agility performance in female TKD athletes. BMI was also a significant predictor, with a non-standardized B of 0.485 (*p* = 0.001) and a standardized Beta of 0.655, indicating that higher BMI is associated with better agility.

In addition, fat percentage (%Fat) showed a significant positive relationship with agility, with a non-standardized B of 1.284 (*p* = 0.015) and a standardized Beta of 0.801, suggesting that higher body fat percentages are associated with better agility test performance. This model explained 74.1% of the variability in TKD-specific agility, with a standard error of estimate of 0.3970. This suggests that BC variables, such as BMI and fat percentage, together with dietary zinc intake, are important predictors of agility test performance in female taekwondo athletes (Table 7).

Collinearity analysis showed no significant problems with the model. Although the body fat percentage had the highest VIF (4.232) and the lowest tolerance (0.236), these values are still within an acceptable range. On the other hand, zinc intake and BMI showed low collinearity, with VIFs of 3.853 and 1.200, respectively. In general, collinearity did not affect the stability of the model or the interpretation of its predictors.

## 4. Discussion

The main aim of this study was to examine the differences in BC, psychological skills, nutrient intake, and physical performance between young high-performance male and female TKD athletes. Additionally, the present study pursued the identification of gender-specific correlations between BC, psychological skills, and nutrient intake with TKD-specific physical performance to understand the factors associated with better TKD performance in young elite TKD athletes. Key findings revealed that males had significantly higher FFM, FM, %Fat, SMM, and EI than females, despite being of similar age and following comparable training and nutrition routines. 

Additionally, males exhibited higher EI and fat intake as well as greater dietary intakes of vitamin A, vitamin C, magnesium, and iron, with a lower intake of vitamin B12, suggesting potential differences in dietary habits between genders. While the total number of kicks in the FSKT_mult_ test was similar for both male and female athletes, males outperformed females in rounds 4 and 5, indicating greater physical performance maintenance. Interestingly, no significant gender differences were observed in psychological attributes related to emotional intelligence, sport success perception, and mental toughness, suggesting comparable psychological preparedness for competition. Lastly, regression models identified body mass as a key predictor of kick count in the FSKT_mult_ for both genders, with calcium intake in males and protein intake in females emerging as additional performance-related factors. These findings highlight the importance of an integrated approach to training, nutrition, and psychological preparation in optimizing TKD performance. Specifically in young elite TKD athletes, performance optimization may require the implementation of gender-specific training and nutrition strategies, emphasizing body mass control and calcium intake for males and protein intake for females.

Nutritional intake analyses demonstrated that males had higher EI and greater consumption of micronutrients, while females showed lower fat intake. Since all participants in this study followed similar dietary habits and comparable training regimens, particularly during the analysis period, the higher nutritional intake of these variables in males is likely attributed to their greater SMM compared to female TKD athletes. However, despite these gender-specific nutritional differences, no significant gender differences were observed in psychological skills, and the performance differences were subtle, with males outperforming females in the later rounds of the FSKT_mult_, but with no significant differences in total kick count of this test or agility performance measured with the TSAT. These findings underscore the complex interplay between BC, nutrition, and performance in young TKD athletes. Regression analyses offer insights into this interplay, indicating that TKD performance is a complex construct with different contributing factors for male and female athletes. While BC, calcium intake, and mental toughness (constancy) were key performance predictors in males, body weight, protein intake, emotional regulation, and FFM played a role in female performance outcomes.

The main outcomes of this study suggest that physical performance outcomes in young elite TKD athletes may be influenced by a combination of factors, reinforcing the need for individualized training, psychological conditioning, and nutrition strategies to optimize long-term athletic success. For instance, significant gender differences were observed in FFM, FM, and SMM, as said, which were higher in male than in female TKD athletes. These differences are consistent with previous studies indicating that male athletes tend to have higher FFM and muscle mass than female counterparts, which is positively associated with better athletic performance, especially in sports requiring explosive movements such as TKD [56]. The anthropometric differences between male and female TKD athletes may be largely influenced by steroid hormones.

Although hormonal levels were not measured in this study, male athletes likely had higher serum testosterone concentrations than females, as evidence shows that circulating testosterone concentrations in men exceed 15-fold that of women at any age [57]. Given testosterone’s role in promoting protein synthesis and muscle mass development [58], it is reasonable to speculate that the observed differences in FFM and SMM are a result of sex-specific hormonal profiles. Indeed, testosterone is a key anabolic steroid hormone, which exerts its effects primarily through androgen receptors (ARs), highly expressed in skeletal muscle tissue. Additionally, females likely have higher estrogen levels [57], which are known to influence fat metabolism and storage. Interestingly, in our study, females had lower FM levels, likely associated with their lower dietary fat intake. This suggests that hormonal regulation may play a dominant role in morphological attributes, but they may be partially altered by nutritional decisions. Nevertheless, both male and female TKD athletes maintain low body fat percentages, likely at levels that do not impede the explosive movements essential for TKD performance [59]. Additionally, their dietary fat intakes were low but remained within the recommended range of 20% to 35% of total daily calories, ensuring adequate hormone production in young athletes [57]. Considering the critical role of BC in TKD performance, sex hormone influences should be carefully considered when training young athletes, as males may have a physiological advantage in muscle development [60], while females may be more predisposed to fat accumulation.

Given the importance of maintaining an optimal body mass, periodic monitoring of both muscle development and fat accumulation is essential to ensure that athletes achieve a balanced BC that supports peak performance while minimizing unnecessary weight gain. These findings highlight the importance of BC in TKD performance, emphasizing the role of muscle mass in power generation while ensuring that low body fat levels do not compromise athletic explosiveness. Understanding these gender-specific physiological differences can aid in developing targeted training and nutrition strategies to further optimize performance and long-term athletic development in young TKD athletes. Last, future research should investigate mRNA expression of ARs in young and adult TKD athletes under basal and post-exercise conditions to better understand its role in muscle development, gender differences, and potential long-term impacts on athletic performance [61]. Indeed, recent evidence suggests that androgen signaling, specifically via ARs, plays a pivotal role in skeletal muscle adaptation to both age and physical training. Studies have shown differential AR mRNA expression between young and adult males, under both basal and post-exercise conditions [60,61,62]. Notably, exercise appears to modulate AR expression, potentially contributing to training-induced muscle hypertrophy. This is especially relevant in the context of young elite athletes like those studied here, where muscle development is crucial to performance. Furthermore, age-related declines in AR levels have been associated with sarcopenia [63] and are evident in skeletal muscle biopsies of older individuals compared to their younger counterparts [64]. All this underscores the importance of considering steroid hormone pathways, particularly testosterone and AR-mediated signaling, when evaluating gender-specific differences in BC and athletic performance. While, as previously mentioned, the present study did not assess hormonal markers directly, future research should explore the interplay between circulating sex hormones, AR expression, nutrition habits, and performance metrics in adolescent athletes to better understand the biological basis of gender disparities in sports performance.

Young male TKD athletes had higher dietary intakes of energy and key micronutrients than their female counterparts, such as vitamin A, magnesium, and iron. The higher EI of male TKD athletes is likely related to their greater muscle mass, which increases metabolic and muscle recovery requirements after exercise [65]. Iron, in particular, plays a crucial role in the formation of hemoglobin and myoglobin, essential molecules for the transport and storage of oxygen during exercise, which supports performance in endurance and power sports [66]. On the other hand, females had lower iron intakes, which could potentially lead to a higher risk of iron deficiency, especially during periods of high exercise volume or intensity or during menstruation, which could affect oxygen transport and muscle recovery [67]. This finding is consistent with research suggesting that up to 30% of female athletes may be iron deficient, negatively impacting their physical performance [68]. Although blood samples were not collected in this study to directly assess iron stores, the findings suggest that iron intake and blood iron levels should be carefully monitored in young TKD athletes, particularly in female athletes, due to their increased susceptibility to iron deficiency and its potential impact on performance and recovery. Regarding macronutrient dietary intake, no significant differences were observed in protein or CHO consumption rates between males and females when normalized per kg of body mass. However, females exhibited a lower fat intake rate, which could impact both recovery and performance in activities requiring repeated efforts. Fat intake is, indeed, a crucial energy source for prolonged, moderate-intensity exercise and contributes to recovery by supporting hormonal balance and cellular repair processes [69].

Therefore, insufficient fat intake might limit the energy reserves available for sustained athletic performance and impair overall recovery efficiency. In addition, the findings of a negative association between fat intake and TKD-specific performance in females suggest that this small difference may have already affected metabolic efficiency. Recent studies suggest that the quality of fat consumed (e.g., unsaturated versus saturated fats) may have an important influence on exercise performance and BC [3]. Exercise practitioners working with young elite TKD athletes should closely monitor iron intake to prevent deficiencies that may impact oxygen transport, endurance, and recovery, especially during high-intensity training periods or menstruation. Additionally, ensuring adequate fat intake with a focus on quality sources can support energy metabolism, hormonal balance, and overall performance, reinforcing the need for individualized nutrition strategies tailored to each athlete’s physiological demands. On the other hand, it seems that young male TKD athletes are less subject to dietary deficits, at least within the age range of participants in this study (i.e., 12–14 years).

Regarding psychological skills, the study found no significant gender differences in the subscales of the tests employed, including emotional intelligence and self-regulation. These results suggest that both male and female athletes have comparable mental skills to cope with the psychological demands of TKD. However, the study observed that psychological factors like mental toughness (specifically confidence and control) may still play a role in overall performance, although no significant gender differences were found in these parameters. This is because mentally tough athletes experience lower levels of cognitive and somatic anxiety and higher self-confidence before competitions [70]. Previous studies suggest that mental toughness and confidence are associated with enhanced sports performance, particularly in high-stress situations, and could be key areas for further development in training programs [71]. Specifically for TKD, a low level of anxiety and neuroticism and a high level of extroversion were found as psychological traits in Olympic TKD competitors [72]. These traits may differ from those observed in other combat sports, suggesting a distinct psychological profile specific to high-performance TKD athletes. In this regard, the study by Nabilpour et al. [48] showed that there is a significant relationship between the control subscale (mental toughness scale) and relative peak power ability. Furthermore, a high level of mental toughness not only motivates athletes to compete but also fosters a sense of control, which can enhance their performance and task execution. Overall, combat sports athletes of higher levels are more intrinsically motivated to experience emotional stimulation, which is a key aspect of their success [73].

In terms of physical performance, the results showed no significant differences between genders in the total kick count of the FSKT_mult_ (Table 4). This suggests that while males may perform better in specific physical tests requiring more explosive movements [74], both genders demonstrated similar endurance and technique, possibly due to the similar nature of TKD training across genders. However, round 4 and round 5 of the FSKT_mult_ revealed significant differences between males and females, with males outperforming females. These findings are consistent with other research that shows that males tend to have better performance in tests of power and strength, partly due to higher muscle mass [75]. The TSAT test did not demonstrate significant gender differences, suggesting that both male and female athletes may exhibit comparable agility despite the differences in other physical attributes. These outcomes highlight the importance of developing fatigue resistance and sustained kicking power in male and female TKD athletes through targeted strength and endurance training, ensuring they can maintain high-intensity performance in later rounds of competition, where physical demands peak. At this age, most gender-specific differences in TKD performance have not yet fully emerged.

Regression models showed that in males, body mass and dietary calcium intake were positively associated with the total number of kicks in the FSKT_mult_, whereas constancy showed a negative association. In females, body mass and protein intake were positively associated with the number of kicks, whereas thiamine intake, FFM, and fat intake were negatively associated with performance in the FSKT_mult_. Physiologically, higher body weight, reflecting greater muscle mass, may contribute to greater strength and power production, which is essential for the execution of fast and powerful kicks in TKD [76]. Adequate calcium intake is essential for muscle contraction and nerve transmission, which may explain its positive association with performance [76]. On the other hand, a high level of endurance, which implies high mental stamina, can lead to increased physical performance in young male TKD athletes.

In females, protein intake supports muscle synthesis and recovery, facilitating better performance in repetitive, high-intensity activities. However, excessive intakes of thiamine and fat may be associated with nutritional imbalances that adversely affect performance [77]. FFM, while generally beneficial, may not translate into improved performance if not accompanied by adequate neuromuscular coordination [78]. A study suggested that reducing fat mass, when combined with proper physical training and dietary planning, can enhance lower limb muscle power in TKD athletes, optimizing explosive movements and overall performance [79]. Effective emotional management allows for better concentration and control during competition, thus improving technical execution [80]. In light of these data, performance optimization in male TKD athletes should focus on maintaining muscle mass and ensuring adequate calcium intake for strength and endurance while also balancing mental stamina to sustain performance over multiple rounds. Female athletes, on the other hand, should prioritize protein intake for muscle recovery and strength while managing fat and thiamine intake to avoid nutritional imbalances that could hinder performance, emphasizing neuromuscular coordination and emotional control to enhance technical execution in competition.

This study has several limitations that should be considered when interpreting the results. The sample size, although sufficient to detect significant associations, was too small to generalize the findings to larger populations of TKD athletes. On the other hand, while this study was specifically conducted with elite competitive TKD athletes who had prior experience in international competitions, the implications of the findings extend beyond this specific group. Given Iran’s prominent position in the international TKD arena, especially within junior categories, as well as its abundance of world-class athletes, the results may provide a valuable framework for other practitioners and coaches in the sport. In addition, specific hormonal factors such as menstrual cycles in women, which may have a relevant effect on BC, energy metabolism, and physical performance, were not included. Another limitation is the cross-sectional study design and the lack of longitudinal measurements, which precludes the assessment of changes in the variables analyzed over time or in response to specific interventions. Despite these limitations, the current study has important strengths. The integration of multiple variables—BC, dietary intake, and psychological skills—provides a holistic perspective on the performance of TKD athletes, which is rare in sports research. In addition, the inclusion of regression analysis allows the identification of key relationships between these variables and their impact on performance, providing a sound basis for the development of personalized training and nutritional strategies.

## 5. Conclusions

In summary, this study highlights the multifaceted nature of TKD performance, demonstrating that BC, nutrition, and psychological attributes play essential but interrelated roles in physical performance among young elite TKD athletes. Key findings indicate that males exhibited greater muscle mass and EI, which correlated with better performance in later rounds of the FSKT_mult_, while females showed lower fat intake and different nutrient-performance relationships, emphasizing the need for individualized nutritional strategies.

Despite gender-based physiological differences, no significant disparities were observed in psychological attributes, suggesting that both male and female athletes exhibit comparable mental resilience to compete in TKD competitions. Notably, this study utilized a high-performance sample, with athletes who had previously competed at the World Taekwondo Championships, earning multiple medals. This elite-level participation enhances the real-world applicability of the findings, making them highly relevant to practitioners working with elite TKD athletes. The results suggest that training and dietary interventions should be tailored to the specific needs of male and female athletes, ensuring optimal physical conditioning, psychological readiness, and dietary adequacy to enhance competitive performance. Ultimately, this holistic approach—considering physical, nutritional, and psychological factors—provides valuable insights for coaches, nutritionists, and sport scientists, reinforcing the importance of integrated strategies to maximize athletic potential in young TKD athletes preparing for high-level competitions.

## Figures and Tables

**Table 1:** Anthropometric and body composition variables in young male and female taekwondo athletes.

Variables	Males		Females		*p*-Value	ES
	Mean	SD	Mean	SD		
Height (cm)	164.9	11.8	160.5	13.4	0.315	0.3
Body mass (kg)	56.4	9.5	52.1	8.3	0.170	0.5
BMI (kg/m^2^)	20.6	1.0	20.1	1.1	0.208	0.4
FFM (kg)	42.8	2.9	36.3	1.6	<0.001	2.7
FM (kg)	3.5	0.7	2.9	0.7	0.009	1.0
%Fat	6.3	0.4	5.6	0.5	<0.001	1.7
SMM (kg)	31.1	2.2	28.2	1.6	<0.001	1.5

Data presented as mean (standard deviation, SD). Abbreviations: BMI: Body Mass Index (kg/m^2^); ES: Effect Size; FFM: Fat-Free Mass (kg); %Fat: Fat Mass (percentage); SMM: Skeletal Muscle Mass (kg). Male and female data were compared using independent *t*-tests to assess statistical differences between groups. ES is Cohen’s d.

**Table 2:** Psychological skills and their subscales in young male and female taekwondo athletes.

Variables	Males		Females		*p*-Value	ES
	Mean	SD	Mean	SD		
Emotional Intelligence						
Evaluation of Others’ Feelings	18.4	1.8	18.2	1.6	0.795	0.1
Evaluation of Own Feelings	19.0	1.6	19.4	1.5	0.462	−0.3
Self-Regulation	19.6	1.2	19.7	1.3	0.875	−0.1
Social Skills	18.4	1.4	18.9	1.8	0.370	−0.3
Employing Emotion	27.9	3.5	28.6	1.5	0.509	−0.2
Optimism	16.6	1.6	17.1	1.4	0.313	−0.4
Sport success						
Flow state	23.6	4.7	23.4	4.9	0.880	0.1
Technique	20.6	2.1	20.3	1.7	0.671	0.1
Sensitivity to Error	23.6	2.6	24.7	3.3	0.241	−0.4
Commitment	28.1	2.1	28.1	2.2	0.964	0.0
Achievement	25.6	2.8	25.5	2.6	0.901	0.0
Mental toughness						
Confidence	14.1	3.0	13.3	2.6	0.421	0.3
Control	5.9	1.6	5.3	1.4	0.239	0.4
Constancy	12.5	1.9	11.7	2.0	0.283	0.4

Data presented as mean (SD). Male and female data were compared using independent *t*-tests to assess statistical differences between groups. Effect size (ES) is Cohen’s d.

**Table 3:** Nutrient intake among young male and female taekwondo athletes.

Variables	Males		Females		*p*-Value	ES
	Mean	SD	Mean	SD		
Protein (g/kg)	1.1	0.1	1.1	0.2	0.591	−0.2
CHO (g/kg)	4.0	0.8	3.9	0.7	0.678	0.1
Fat (g/kg)	1.4	0.3	1.1	0.1	0.003	1.1
EI (kcal/kg)	32.4	4.6	29.3	3.1	0.032	0.8
Vitamin A (µg/d)	1386.8	300.9	921.4	323.8	<0.001	1.5
Vitamin C (mg/d)	102.0	27.4	80.4	20.0	0.014	0.9
Vitamin E (mg/d)	3.4	1.0	3.5	0.9	0.854	−0.1
Vitamin D (mg/d)	4.9	1.1	4.9	0.9	0.988	0.0
Vitamin B12 (µg/d)	5.5	1.5	6.7	1.2	0.017	−0.9
Folate (µg/d)	168.0	26.0	172.0	17.0	0.588	−0.2
Thiamine (mg/d)	1.4	0.3	1.4	0.3	0.987	0.0
Riboflavin (mg/d)	2.0	0.3	2.0	0.3	0.903	0.0
Niacine (mg/d)	3.3	0.9	3.3	0.8	0.867	0.1
Magnesium (g/d)	318.7	45.1	272.0	41.7	0.004	1.1
Zinc (mg/d)	11.4	2.2	10.1	2.9	0.115	0.6
Calcium (mg/d)	1203.0	191.0	1184.0	190.0	0.780	0.1
Iron (mg/d)	17.0	2.4	14.4	3.6	0.017	0.9
Fiber (g/d)	12.0	3.1	12.8	1.8	0.391	−0.3

Notes: Data presented as mean (SD). CHO: Carbohydrates; EI: Energy Intake. Male and female data were compared using independent *t*-tests to assess statistical differences between groups. Effect size (ES) is Cohen’s d.

**Table 4:** Physical performance in young male and female taekwondo athletes undergoing the Frequency Speed of Kick Test (FSKT_mult_) and the Taekwondo-Specific Agility Test (TSAT).

Variables	Males		Females		*p*-Value	ES
	Mean	SD	Mean	SD		
Round 1 (n)	21.8	1.2	21.3	1.8	0.370	0.3
Round 2 (n)	21.0	1.2	20.6	1.6	0.405	0.3
Round 3 (n)	19.8	1.6	19.8	1.5	0.925	0.0
Round 4 (n)	19.4	1.1	18.6	1.4	0.048	0.7
Round 5 (n)	18.2	1.0	17.2	1.0	0.007	1.0
Total Number of Kicks (n)	100.2	4.6	97.5	5.9	0.139	0.5
Kick Decrement Index (%)	8.1	0.3	8.0	0.3	0.469	0.3
TSAT time (s)	8.0	10.6	6.1	0.8	0.489	0.2

Notes: Data presented as mean (SD). Male and female data were compared using independent *t*-tests to assess statistical differences between groups. Effect size (ES) is Cohen’s d.

**Table 5:** Best-fit regression model to estimate total kick numbers (dependent variable) in young male taekwondo athletes.

Model	Non-Standardized Coefficients		Standardized Coefficients	T	Sig.	Summary of the Model		Collinearity Statistics	
	B	Standard Error	Beta			R^2^	Adjusted R^2^	Tolerance	VIF
(Constant)	78.835	2.656		29.687	<0.001	0.911	0.893		
Body mass	0.417	0.044	0.901	9.484	<0.001			0.660	1.514
Constancy	−0.831	0.213	−0.358	−3.899	0.001			0.707	1.414
Calcium intake	0.007	0.002	0.284	2.899	0.011			0.619	1.616

**Table 6:** Best-fit regression model to estimate the total kick numbers in the FSKT_mult_ (dependent variable) in young female taekwondo athletes.

Model	Non-Standardized Coefficients		Standardized Coefficients	T	Sig.	Summary of the Model		Collinearity Statistics	
	B	Standard Error	Beta			R^2^	Adjusted R^2^	Tolerance	VIF
(Constant)	141.943	14.918		9.515	<0.001	0.981	0.967		
Body mass	0.966	0.093	1.372	10.433	<0.001			0.137	7.308
Thiamine intake	−6.296	0.982	−0.327	−6.415	<0.001			0.908	1.101
Protein intake	6.748	2.077	0.171	3.249	0.012			0.851	1.175
FFM	−2.551	0.474	−0.683	−5.386	0.001			0.147	6.797
Fat intake	−19.049	3.966	−0.269	−4.803	0.001			0.752	1.331
Employing Emotion	0.676	0.211	0.174	3.208	0.012			0.808	1.238

**Table 7:** Best-fit regression model to estimate time in the Taekwondo-Specific Agility Test performance (dependent variable) in female taekwondo athletes.

Model	Non-Standardized Coefficients		Standardized Coefficients	T	Sig.	Summary of the Model		Collinearity Statistics	
	B	Standard Error	Beta			R^2^	Adjusted R^2^	Tolerance	VIF
(Constant)	−7.720	3.449		−2.239	0.047	0.796	0.741		
Zinc intake	−0.309	0.071	−1.164	−4.357	0.001			0.260	3.853
BMI	0.485	0.110	0.655	4.395	0.001			0.833	1.200
%Fat	1.284	0.448	0.801	2.862	0.015			0.236	4.232

## Data Availability

The datasets used and/or analyzed during the current study are available from the corresponding author upon reasonable request.
